# Genomic clustering of fitness‐affecting mutations favors the evolution of chromosomal instability

**DOI:** 10.1111/eva.12717

**Published:** 2018-10-11

**Authors:** Yevgeniy Raynes, Daniel M. Weinreich

**Affiliations:** ^1^ Department of Ecology and Evolutionary Biology, Center for Computational Molecular Biology Brown University Providence Rhode Island

**Keywords:** carcinogenesis, chromosomal instability, indirect selection, mutator

## Abstract

Most solid cancers are characterized by chromosomal instability (CIN)—an elevated rate of large‐scale chromosomal aberrations and ploidy changes. Chromosomal instability may arise through mutations in a range of genomic integrity loci and is commonly associated with fast disease progression, poor prognosis, and multidrug resistance. However, the evolutionary forces promoting CIN‐inducing alleles (hereafter, CIN mutators) during carcinogenesis remain poorly understood. Here, we develop a stochastic, individual‐based model of indirect selection experienced by CIN mutators via genomic associations with fitness‐affecting mutations. Because mutations associated with CIN affect large swaths of the genome and have the potential to simultaneously comprise many individual loci, we show that indirect selection on CIN mutators is critically influenced by genome organization. In particular, we find strong support for a key role played by the spatial clustering of loci with either beneficial or deleterious mutational effects. Genomic clustering of selected loci allows CIN mutators to generate favorable chromosomal changes that facilitate their rapid expansion within a neoplasm and, in turn, accelerate carcinogenesis. We then examine the distribution of oncogenic and tumor‐suppressing loci in the human genome and find both to be potentially more clustered along the chromosome than expected, leading us to speculate that human genome may be susceptible to CIN hitchhiking. More quantitative data on fitness effects of individual mutations will be necessary, though, to assess the true levels of clustering in the human genome and the effectiveness of indirect selection for CIN. Finally, we use our model to examine how therapeutic strategies that increase the deleterious burden of genetically unstable cells by raising either the rate of CIN or the cost of deleterious mutations affect CIN evolution. We find that both can inhibit CIN hitchhiking and delay carcinogenesis in some circumstances, yet, in line with earlier work, we find the latter to be considerably more effective.

## 1 INTRODUCTION

Genomic instability is a hallmark of carcinogenesis and may account for much of the extensive genetic heterogeneity of solid tumors (Bedard, Hansen, Ratain, & Siu, 2013; Burrell, McGranahan, Bartek, & Swanton, 2013; Marusyk, Almendro, & Polyak, 2012; Salk, Fox, & Loeb, 2010; Vogelstein et al., 2013). Genomic instability can be categorized as nucleotide instability (NIN), microsatellite instability (MSI), or chromosomal instability (CIN). Nucleotide instability manifests as an elevated rate of single nucleotide alterations and arises from defects in the nucleotide and base excision repair pathways (Pikor, Thu, Vucic, & Lam, 2013). Microsatellite instability is associated with an elevated rate of nucleotide mismatches and short deletions and insertions (Boland & Goel, 2010; Thibodeau, Bren, & Schaid, 1993) and usually results from defects in the mismatch repair system (Vilar & Gruber, 2010). Most commonly, solid tumors exhibit chromosomal instability—an elevated rate of large chromosomal aberrations, such as somatic copy number alterations (SCNAs) and aneuploidies (Bakhoum & Compton, 2012; Hanahan & Weinberg, 2011; Heng et al., 2013). Unlike NIN or MSI, molecular mechanisms of CIN—especially in sporadic cancers—remain poorly defined. Mutations in a number of different pathways have been implicated in CIN, including, among others, mitotic checkpoints (Cahill et al., 1998), chromatid cohesion (Solomon et al., 2011), and double‐strand break repair (Lord & Ashworth, 2012). Chromosomal instability has been observed in most high mortality cancers including colon (Fearon, 2011; Lengauer, Kinzler, & Vogelstein, 1998), breast (Kwei, Kung, Salari, Holcomb, & Pollack, 2010), and lung (Masuda & Takahashi, 2002) and has been linked with poor prognosis (Carter, Eklund, Kohane, Harris, & Szallasi, 2006; McGranahan, Burrell, Endesfelder, Novelli, & Swanton, 2012; Walther, Houlston, & Tomlinson, 2008) and multidrug resistance (Lee et al., 2011). Despite its clinical importance, the evolutionary forces favoring the emergence of CIN and its role in cancer progression have long been a subject of debate in the literature and remain poorly understood (Cahill, Kinzler, Vogelstein, & Lengauer, 1999; Datta, Gutteridge, Swanton, Maley, & Graham, 2013; Loeb, 2011; Michor, 2005; Negrini, Gorgoulis, & Halazonetis, 2010; Nowak et al., 2002).

Current theories for the evolution of CIN, and, in fact, the evolution of genomic instability in general, usually invoke two distinct, yet not mutually exclusive, mechanisms of selection on instability‐inducing mutator alleles. Chromosomal instability mutators may intrinsically raise a neoplastic cell's chances of survival and reproduction, that is, its Darwinian fitness. As a result, CIN mutators may be directly favored by natural selection. For example, it has been suggested that CIN may originate from directly beneficial oncogenic mutations that simultaneously induce genomic instability [the oncogene‐induced DNA replication stress hypothesis] (Halazonetis, Gorgoulis, & Bartek, 2008; Negrini et al., 2010).

Alternatively, mutators may be favored not for their own intrinsic effects on a cell's fitness but through genetic association with intrinsically beneficial mutations elsewhere in the genome. In other words, CIN mutators may evolve via so‐called indirect selection by hitchhiking (Smith & Haigh, 1974) with selectively favored oncogenic and tumor‐suppressing mutations they generate (Loeb, 2001; Sprouffske, Merlo, Gerrish, Maley, & Sniegowski, 2012). Indirect selection on alleles that increase the genomic mutation rate (e.g., CIN‐inducing mutators) is expected to be particularly effective in asexual populations, such as cancers, in which the genetic association between mutators and beneficial mutations can never be disrupted by recombination. In fact, numerous theoretical and experimental studies in microbes have already shown that mutators may spread through non‐recombining populations by hitchhiking if beneficial mutations are readily available (reviewed in: Raynes & Sniegowski, 2014; Sniegowski, Gerrish, Johnson, & Shaver, 2000).

Importantly, while genomic instability may increase the rate of beneficial mutations, it necessarily also increases the rate of deleterious mutations, which are generally more common (Cahill et al., 1999). However, alleles that elevate the point mutation rate (i.e., NIN‐ or MSI‐inducing mutators) generate DNA changes confined to only a few nucleotides. As a result, beneficial and deleterious mutations are likely introduced independently from each other by separate mutational events. Thus, while such mutators may be frequently lost to selection against the increased load of deleterious mutations, they may also occasionally expand within a neoplasm by hitchhiking with a rare beneficial mutation. In contrast, CIN mutators generate large‐scale SCNAs that are not confined to single loci. Instead, SCNAs may disrupt many neighboring loci, thereby simultaneously introducing both beneficial and deleterious changes. For example, a single SCNA may delete a tumor suppressor and a neighboring housekeeping gene. Genetic linkage between the relatively rare beneficial loci and the more common deleterious ones may drastically limit the availability of SCNAs with net beneficial effects (needed to facilitate CIN hitchhiking) and, thus, inhibit CIN evolution.

We have hypothesized that indirect selection could, nevertheless, favor CIN given a spatial organization of the genome that minimizes co‐occurrence of beneficial and deleterious loci in SCNAs. Specifically, we wanted to test the hypothesis that CIN would be favored by indirect selection in genomes in which either beneficial or deleterious loci were spatially clustered along the chromosome. To do so, we developed an individual‐based stochastic population model of clonal evolution in spatially organized genomes. In simulation, we investigated the effect of the spatial distribution of beneficial and deleterious loci on CIN evolution and cancer progression. We also tested the effectiveness of therapeutic strategies that aim to raise the deleterious costs of CIN in order to inhibit CIN evolution. Finally, we examined the spatial distributions of candidate human oncogene and tumor suppressor loci (identified in Davoli et al., 2013) for evidence of spatial clustering that could facilitate indirect selection for CIN in real cancers.

## 2 METHODS

### 2.1 Stochastic simulations

To model the evolutionary progression of a neoplastic cell population to cancer, we developed and simulated an individual‐based computational model of clonal evolution based on the earlier work of Beerenwinkel et al. (2007) and Datta et al. (2013). Like these earlier studies, we employed a Wright–Fisher model (Ewens, 2004), in which a population of neoplastic cells evolves in discrete, non‐overlapping generations with the probability of each cell's reproduction being proportional to its relative fitness. As in earlier studies, cells in the model could acquire beneficial and deleterious fitness‐affecting mutations upon reproduction. Unlike these earlier studies, however, we also allowed for the evolution of CIN by adding SCNA‐type mutations (described below) and introducing CIN mutator alleles.

As in the work of Beerenwinkel et al. (2007) and Datta et al. (2013), simulations here start with a tumor population of initial size *N*_0_ = 10^6^ cells and end when the tumor develops into a cancer. Following Datta et al. (2013), we defined cancer as a tumor in which 10% of all cells have acquired at least 20 beneficial mutations.

The total size of the neoplastic population is constrained to grow exponentially at a rate proportional to the average fitness of the population. The size of the population at generation *t* + 1 is defined as $N_{t+1}=N_{t}\cdot \left(1+\overset{¯}{w}\beta \right)$, where $\overset{¯}{w}$ is the average fitness of the tumor population (see below) and *β* is a constant that governs the rate of population growth; as in Datta et al. (2013), we set *β* = 0.0016.

Neoplastic cells can acquire single‐locus mutations that either change fitness or induce CIN. Chromosomal instability‐inducing mutations result in a mutator phenotype which allows cells to generate SCNA mutations (described below). In order to explicitly explore the role of indirect selection and genetic hitchhiking in the evolution of CIN, CIN‐inducing mutations are assumed to have no intrinsic direct effect on a cell's fitness. Furthermore, a single CIN mutator mutation is assumed to be sufficient for the mutator phenotype; additional CIN mutations have no effect on the mutation rate and so cannot affect the dynamics of the mutator lineage.

Among the fitness‐affecting mutations, beneficial mutations increase a cell's fitness by *s_ben_* while deleterious mutations decrease a cell's fitness by *s*_del_. Since *s*_ben_ and *s*_del_ are held constant, fitness of a cell with *x* beneficial mutations and *y* deleterious mutations can be computed as $w_{\mathrm{xy}}=1+x\cdot s_{\mathrm{ben}}-y\cdot s_{\mathrm{del}}$. Correspondingly, if *f_xy_* is the fraction of cells with *x* and *y* beneficial and deleterious mutations, respectively, in a tumor, then the average fitness of the tumor population is $\overset{¯}{w}=\sum_{x}\sum_{y}f_{\mathrm{xy}}w_{\mathrm{xy}}$. Unlike in the earlier work (Beerenwinkel et al., 2007; Datta et al., 2013), the effect of multiple mutations in our model is additive rather than multiplicative. Note that there is little difference between additive and multiplicative fitness for genotypes containing a small number of mutations. (Algebraically, ${\left(1+s_{\mathrm{ben}}\right)}^{x}{\left(1-s_{\mathrm{del}}\right)}^{y}\approx 1+x\cdot s_{\mathrm{ben}}-y\cdot s_{\mathrm{del}}$ for small *x* and *y*.) However, for genotypes containing many mutations (like some CIN genotypes in our model), multiplicative fitness, which increases exponentially with the number of mutations, results in unrealistically high values.

The tumor population is composed of genetic lineages of neoplastic cells defined by the counts of fitness‐affecting and CIN mutator mutations they carry. As per the Wright–Fisher model, the size of a lineage with *x* beneficial mutations and *y* deleterious mutations at generation *t* + 1 is drawn from a multinomial distribution with expectation given by $N_{t+1}f_{\mathrm{xy}}w_{\mathrm{xy}}/\overset{¯}{w}$, where $f_{\mathrm{xy}}$ is the frequency of the lineage in generation *t*, and $w_{\mathrm{xy}}/\overset{¯}{w}$ is its relative fitness.

Upon reproduction, every surviving lineage acquires a random number *M*_ben_ of beneficial mutations*, M*_del_ of deleterious mutations, and *M*_CIN_ of mutator mutations drawn from a multinomial distribution with expectations given by *N_i_U*_ben_, *N_i_U*_del_, and *N_i_U*_CIN_, respectively. Here, *N_i_* is the size of the lineage, and *U*_ben_, *U*_del_, and *U*_CIN_ are the per cell rates of beneficial, deleterious, and CIN mutator mutations. Thus, as a result of mutation, a lineage with *x* beneficial and *y* deleterious mutations gives rise to a new lineage with *M*_ben_ individuals carrying *x* + 1 beneficial mutations, a new lineage with *M*_del_ individuals carrying *y* + 1 deleterious mutations, and a third lineage with *M*_CIN_ individuals carrying the CIN mutator allele.

Genetic lineages carrying CIN mutator mutations also acquire a Poisson‐distributed number of SCNAs, *M*_SCNA_ with mean *N_i_U*_SCNA_, where *U*_SCNA_ is the per cell rate of SCNA production in CIN mutators. Each new SCNA mutation contains a randomly generated number of beneficial, *B*_SCNA_, and a randomly generated number of deleterious mutations, *D*_SCNA_. Each new SCNA mutation in a lineage with *x* beneficial and *y* deleterious mutations, thus, gives rise to a new lineage with a single individual carrying *x* + *B*_SCNA_ beneficial and *y + D*_SCNA_ deleterious mutations.

To model spatial clustering of fitness‐affecting loci, we use discrete distributions with a constant mean number of mutations (*µ*) but increasing variance (*σ* ^2^) to draw the values of *B*_SCNA_ and *D*_SCNA_ for each new SCNA. Thus, we assume the infinite sites model for new SCNAs. Note that while the available supply of large chromosomal aberrations, such as aneuploidies, in real cancers is relatively small, shorter SCNAs will be plentiful. In fact, the supply of SCNAs containing about 100 loci (~0.5% of the human genome) like the ones in our model should be extremely high, justifying our assumption of the infinite sites model. Furthermore, consider that in our model a tumor samples and substitutes only an infinitesimal fraction of the available SCNA distribution, similar to real cancers that have been shown to contain a median number of only about 20 SCNAs (Beroukhim et al., 2010).

**Figure 1:**
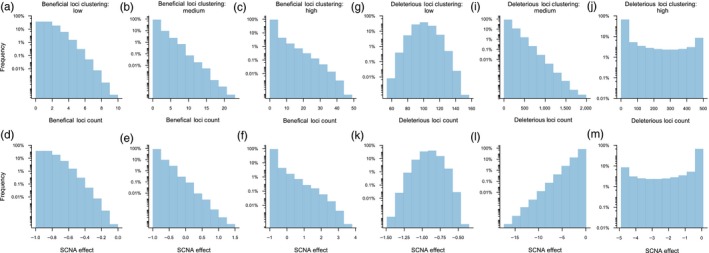
Spatial clustering of fitness‐affecting loci in the model. Beneficial loci of effect *s*_ben_ = 0.1 were distributed across SCNAs using either Poisson (a; low clustering), geometric (b; intermediate clustering), or beta‐binomial (C; high clustering) distributions with *µ* = 1. Deleterious loci of effect *s*_del_ = 0.01 were distributed using the Dirac delta function with *µ* = 100. These distributions of beneficial loci produced the distributions of SCNA fitness effects shown in panels d, e, and f, respectively, calculated as $w_{\mathrm{SCNA}}=B\cdot s_{\mathrm{ben}}-D\cdot s_{\mathrm{del}}$ (where *B* and *D* are the counts of beneficial and deleterious mutations, respectively). Likewise, deleterious mutations were distributed across SCNAs using Poisson (g; low clustering), geometric (i; intermediate clustering), or beta‐binomial (j; high clustering) distributions with *µ* = 100. Beneficial loci were distributed using the Dirac delta function with *µ* = 1. These distributions of deleterious loci produced the distributions of SCNA fitness effects shown in panels k, l, and m, respectively, calculated as above. See Methods for distribution details

Because we assume the infinite sites model for new SCNAs, spatially organized genomes are not explicitly modeled, which allows increased computational efficiency. Instead, clustering of loci in the model is controlled by the variance in the distributions of *B*_SCNA_ and *D*_SCNA_. As can be seen from Figure 1, distributions of *B*_SCNA_ and *D*_SCNA_ characterized by lower variance result in less clustering of fitness‐affecting loci across potential SCNAs with most SCNAs containing relatively similar numbers of loci. On the other hand, higher variance in either *B*_SCNA_ or *D*_SCNA_ distributions produces more clustering in fitness‐affecting loci across potential SCNAs with relatively few SCNAs containing large clusters of loci and many SCNAs without any.

The model was parameterized as follows. Based on earlier theoretical studies of Beerenwinkel et al. (2007), Bozic et al. (2010), Datta et al. (2013), and McFarland, Korolev, Kryukov, Sunyaev, and Mirny (2013), we set *U*_ben_ = 10^‐5^. Because estimates of the deleterious mutation rate and effects are limited, we set *s*_del_ = 0.01 and we assumed that deleterious mutations outnumber the beneficial ones one hundred‐fold and set *U*_del_ = 10^–3^. Note that higher *U*_del_ or *s*_del_ would raise the deleterious load of SCNAs, reinforcing the importance of genomic clustering in promoting CIN; on the other hand, lower *U*_del_ or *s*_del_ would lessen the role of genomic clustering in facilitating CIN evolution. *U*_CIN_ has no effect on the role of genomic clustering and was set at *U*_CIN_ = 10^‐5^. We set *U*_SCNA_ = 0.01 SCNA mutations per cell per generation after Lengauer, Kinzler, and Vogelstein (1997) in lineages carrying CIN mutators. Finally, we set *s*_ben_ = 0.1, based on the estimates of beneficial effects of ~0.004 to ~0.6 obtained from earlier theoretical studies (Beerenwinkel et al., 2007; Bozic et al., 2010; McFarland, Mirny, & Korolev, 2014).

The mean number of mutations (*µ*) of all *B*_SCNA_ distributions is set to 1 (i.e., on average a single beneficial mutation per SCNA), while *µ* of all *D*_SCNA_ distributions is set to 100: The ratio of the two is thus the same as the ratio of *U*_ben_ and *U*_del_ above. To generate *B*_SCNA_ values, we use either a Dirac delta function with a single value (i.e., no clustering, *µ* = 1, *σ* ^2^ = 0), a Poisson distribution (minimal clustering, *µ* = 1, *σ* ^2^ = 1; Figure 1a), a geometric distribution (intermediate clustering, *µ* = 1, *σ* ^2^ = 2; Figure 1b), or a beta‐binomial distribution (high clustering, parameters of the beta distribution: *n* = 50, *α* = 0.1, *β* = 4.9; *µ* = 1, *σ^2^* ≈ 8.98; Figure 1c). Similarly, *D*_SCNA_ is randomly drawn from either a Dirac delta function (*µ* = 100, *σ^2^* = 0), a Poisson distribution (*µ* = 100, *σ^2^* = 100; Figure 1g), a geometric distribution (*µ* = 100, *σ^2^* = 10,100; Figure 1i), or a beta‐binomial distribution (parameters of the beta distribution: *n* = 500, *α* = 0.1, *β* = 0.4; *µ* = 100, *σ* ^2^ ≈ 26,693; Figure 1j). Figure 1 also illustrates how changing the variance in the physical distribution of beneficial and deleterious loci affects the distribution of available SCNA fitness effects (calculated for an SCNA with *B* beneficial and *D* deleterious mutations as $w_{\mathrm{SCNA}}=B\cdot s_{\mathrm{ben}}-D\cdot s_{\mathrm{del}}$). It is important to note that while the expected number of loci in an SCNA is constant for all distributions (100 deleterious plus 1 beneficial), the realized number of loci in an SCNA is not constant unless both *B*_SCNA_ and *D*_SCNA_ are drawn from the Dirac delta function. Consequently, at higher variance in either *B*_SCNA_ or *D*_SCNA_, some SCNAs may comprise more loci than others. Moreover, while we model SCNAs of a given mean size (101 loci), it is certainly not the case that SCNAs of such size are representative of actual SCNAs in cancer (in fact, based on Beroukhim et al. (2010), it is likely that most focal SCNAs comprise fewer loci). We explore these assumptions in the simulation in Supporting Information Figures S1 and S2, respectively, and find that the effect of clustering on CIN evolution is robust to both of them.

Since all simulations end with cancer, to assess the influence of genomic clustering and CIN evolution on carcinogenesis, we calculate the mean waiting time required for a tumor to evolve into cancer. To assess whether CIN mutators are favored by indirect selection or not, we calculate the mean waiting time to CIN establishment. We assume that a mutator mutation becomes established if it reaches the frequency of 10% of the population. We then compare the probability of mutator establishment ($P_{10\%}^{\mathrm{CIN}}$) to the probability of a neutral mutation reaching the frequency of 10% ($P_{10\%}^{\mathrm{neutral}}$).$P_{10\%}^{\mathrm{neutral}}$ is calculated as the frequency (over 10^6^ runs of simulation) at which a neutral mutation appearing at the same rate as the mutator (*U*_CIN_) reaches 10% of the population in control simulations without CIN. Correspondingly, mutators are favored by indirect selection when $P_{10\%}^{\mathrm{CIN}}$> $P_{10\%}^{\mathrm{neutral}}$ and disfavored when $P_{10\%}^{\mathrm{CIN}}$<$P_{10\%}^{\mathrm{neutral}}$ (e.g., Raynes, Wylie, Sniegowski, & Weinreich, 2018).

### 2.2 Genomic analysis

To examine the spatial distribution of the carcinogenic mutations in the human genome, we focused on the candidate oncogene and tumor suppressor loci identified by the TUSON algorithm of Davoli et al. (2013). The TUSON algorithm predicts the likelihood that a given gene acts as a tumor suppressor or an oncogene based on its mutational status in sequenced tumors versus normal tissue. To identify the most reliable mutational parameters for prediction of tumor suppressors and oncogenes, Davoli et al. compiled a data set of ~1,200,000 mutations from more than 8,200 tumor samples of >20 different tumor types and developed 22 different parameters based on the different classes of mutations. The algorithm was tested on three different training sets of known tumor suppressors and oncogenes from the Cancer Gene Census (Futreal et al., 2004). In the original study, TUSON predictions were used to rank every gene in the genome based on its potential as a tumor suppressor or an oncogene (Davoli et al., 2013). For our analysis, we used the top 300 tumor suppressors and 249 oncogenes identified by TUSON in the original study. One of the potential tumor suppressors (C3orf27) from the original top 250 list of Davoli et al. have since been shown to be a long intergenic non‐protein‐coding RNA and was excluded from the present analysis. As in Davoli et al., each potential tumor suppressor and oncogene was assigned a weight, hereafter $w_{\mathrm{Davoli}}$ (to differentiate from fitness notation in our model), calculated as $w_{\mathrm{Davoli}}=T-r$ where *T* is the total number of genes in the respective list (300 for suppressors and 249 for oncogenes) and *r* is the rank of that gene in the list. $w_{\mathrm{Davoli}}$ can thus be thought as proxy for the potential fitness effect of a gene (most potent tumor suppressors and oncogenes have the highest $w_{\mathrm{Davoli}}$ scores).

Then, also as in Davoli et al., we quantified the tumor‐suppressive and oncogenic potentials of human SCNAs based on the density and potency ($w_{\mathrm{Davoli}}$) of tumor suppressors and oncogenes they encompass. For SCNAs the size of a chromosome or chromosome arm we used Chrom and Charm scores provided in the study of Davoli et al. These scores were calculated as the sums of weights ($w_{\mathrm{Davoli}}$) of tumor suppressors or oncogenes contained in each chromosome or a chromosome arm divided by the total number of genes contained in that chromosome or arm. In other words, Chrom and Charm scores represent the potential beneficial effect that SCNAs comprising a given chromosome or a chromosome arm may have on a neoplastic cell's fitness.

For focal SCNAs shorter than a chromosome arm, we employed a sliding window approach. For computational efficiency, we advanced the window from the beginning of each chromosome arm, 10,000 nucleotides at a time, up until the centromere or the end of the chromosome was reached. The score of each window was calculated as the sum of weights of oncogenes or tumors suppressors contained in the window divided by the total number of genes in the window. To quantify spatial clustering of oncogenes and tumor suppressors across chromosomes, chromosome arms, and focal SCNAs, we calculated the variance in the distributions of Chrom, Charm, and focal SCNA scores of each size.

To assess whether human oncogenes and tumor suppressors are clustered more than random, we used a permutation approach. To create each random permutation of tumor suppressors and oncogenes, weight ($w_{\mathrm{Davoli}}$) values from 1 to 300 (for the tumor suppressor distribution) or from 1 to 249 (for the oncogene distribution) were assigned to 300 (or 249) human loci, randomly sampled from the genome. For each such random permutation, we calculated the Chrom, Charm, and focal SCNA scores as above. We then calculated the variance in the distributions of Chrom, Charm, and focal SCNA scores for each permutation. A total of 10,000 permutations of weights were performed resulting in distributions of 10,000 variance values for each size of potential SCNAs. Observed variance in the distribution of Chrom, Charm, and focal scores of all sizes for tumor suppressors and oncogenes was then compared to the null distributions obtained by permutation. Observed variances were considered to be significantly higher than expected if they were greater than 95% of variances sampled by permutation.

Ensemble database, release 84 (Yates et al., 2016), was used to obtain the genomic locations of all tumor‐suppressing and oncogenic genes identified by TUSON as well as the list of 20,837 human genes using the following two gene types to filter the results: protein‐coding genes and TEC (potential protein‐coding genes that require experimental confirmation). Genes on the Y chromosome were excluded from the analysis. The UCSC human genome browser (Karolchik et al., 2004) (Assembly Dec 2013, GRCh/hg38) was used to obtain the lengths of all human chromosomes and the locations of centromeres.

## 3 RESULTS

### 3.1 Spatial clustering of fitness‐affecting loci across SCNAs promotes the evolution of CIN

**Figure 2:**
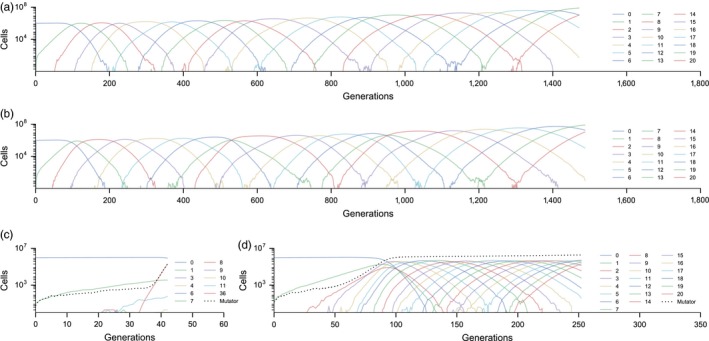
Clonal dynamics in representative simulated populations. Solid lines represent the size of clones with numbers of beneficial mutations indicated. Dashed lines in c and d represent the size of the mutator population within the tumor. (a) Without CIN mutators, (b) with CIN mutators; beneficial and deleterious mutations distributed with no clustering (using the Dirac delta function), (c) with CIN mutators; beneficial mutations distributed with high clustering (using the beta‐binomial distribution) and deleterious loci not clustered, (d) with CIN mutators; deleterious mutations distributed with high clustering (beta‐binomial) and beneficial loci not clustered. See Methods for distribution details. Model parameters: *U*_ben_ = 10^−5^, *U*_del_ = 10^−3^, *U*_CIN_ = 10^−5^ per cell per generation, *s*_del_ = 0.01, *s*_ben_ = 0.1, *U*_SCNA_ = 0.01 SCNA mutations per cell per generation. *µ* for all beneficial loci distributions = 1, *µ* for all deleterious loci distributions = 100

Figure 2 illustrates the dynamics of tumor evolution in representative runs of the simulation with and without CIN mutator loci in the genome (note the difference in x‐axis scale between panels). Consistent with earlier studies (Beerenwinkel et al., 2007; Datta et al., 2013), in the absence of CIN mutators, our model exhibits a “traveling wave” of clonal expansions and contractions (Figure 2a). Clones with increasing numbers of beneficial mutations successively expand and replace less adapted clones until a clone with 20 beneficial mutations appears and expands to 10% of the population (at which point the simulation halts). Importantly, each clonal expansion is driven by only a single additional beneficial mutation.

When CIN mutators are added to the genome, the dynamics of tumor evolution become strongly dependent on the spatial distribution of beneficial and deleterious loci across potential SCNAs. Recall that in our model, CIN mutators themselves do not affect fitness and only experience indirect selection via genetic association with SCNA mutations they generate. When neither beneficial nor deleterious loci are spatially clustered, that is, distributed across SCNAs with no variance, all available SCNAs are deleterious to fitness (Figure 1). As a result, CIN mutators are strongly disfavored by indirect selection and have virtually no effect on cancer progression, which remains indistinguishable from that in genomes without CIN (Figure 2b).

However, when either beneficial or deleterious loci are highly spatially clustered, that is, distributed with the highest possible variance in the genome, cancer progression is significantly accelerated by CIN, albeit in different ways. In genomes with highly clustered beneficial loci, large numbers of beneficial mutations co‐occur in a few SCNAs, resulting in some extremely beneficial SCNAs (Figure 1c,f). Chromosomal instability mutators rapidly increase in frequency by acquiring such SCNAs, which, in turn, allows the tumor to very quickly evolve into cancer by substituting only a few SCNAs. For example, in Figure 2c, a single CIN mutator lineage acquires a cluster of 36 beneficial mutations, which then quickly expands to the threshold frequency of 10%. On the other hand, in genomes with highly clustered deleterious mutations (Figure 1j,m), large numbers of deleterious loci are sequestered into few very harmful SCNAs, which reduces the deleterious load of other available SCNAs. However, spatial clustering of deleterious mutations does not affect the number of beneficial mutations in individual SCNAs. As a result, CIN mutators are still favored by indirect selection but spread by hitchhiking with SCNAs containing fewer than the mean number of deleterious mutations and only a single beneficial mutation. Correspondingly, up to 20 SCNA mutations may substitute in the CIN mutator population before the tumor develops into cancer. Populations with clustered deleterious mutations exhibit very similar traveling wave dynamics as populations without CIN, although the progression is accelerated once CIN mutators become established (Figure 2d).

**Figure 3:**
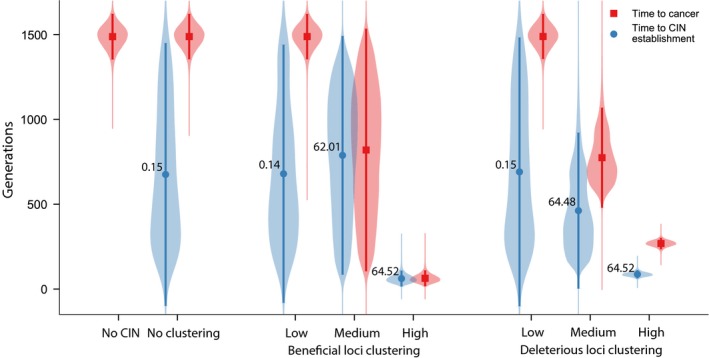
Clustering of fitness‐affecting loci promotes CIN evolution and accelerates cancer development. Waiting time to CIN establishment (blue) and cancer (red) as a function of genomic organization. For genomes with clustered beneficial mutations (*µ = *1), deleterious loci were distributed using the Dirac delta function (*µ* = 100). For genomes with clustered deleterious mutations (*µ* = 100), beneficial loci were distributed using the Dirac delta function (*µ* = 1). The rest of model parameters are as in Figure 2. Circles are mean values calculated over 100,000 runs of simulation (error bars represent ±95% CI, all times are represented with violin plots).$P_{10\%}^{\mathrm{CIN}}/P_{10\%}^{\mathrm{neutral}}$ values next to each time to CIN establishment data point. Mutators are favored by selection when $P_{10\%}^{\mathrm{CIN}}/P_{10\%}^{\mathrm{neutral}}>1$. $P_{\mathrm{est}}^{\mathrm{neutral}}$ ≈ 0.016 (see Methods). See Methods for more distribution details

Figure 3 summarizes the influence of the spatial distribution of fitness‐affecting loci across SCNAs on CIN evolution and the speed of carcinogenesis. As expected from population dynamics seen in Figure 2, cancer progression is slowest in genomes without CIN mutators. Even in genomes with CIN mutators, cancer progression remains unaffected by CIN when both beneficial and deleterious loci are distributed with no or even low clustering across SCNAs. In these genomes, CIN mutators are strongly disfavored by indirect selection ($P_{10\%}^{\mathrm{CIN}}<P_{10\%}^{\mathrm{neutral}}$) and the waiting time to cancer is not significantly different than in genomes without CIN (Figure 3). However, as beneficial and deleterious loci become increasingly clustered, the probability of mutator establishment raises dramatically above that of a neutral mutation ($P_{10\%}^{\mathrm{CIN}}>P_{10\%}^{\mathrm{neutral}}$). In other words, CIN mutators switch from being disfavored to being strongly favored by indirect selection. Correspondingly, as selected loci become increasingly clustered, cancer progression is accelerated, with the waiting time to cancer minimized in genomes with the most clustered loci.

Note that in genomes with clustered beneficial loci, the waiting time to CIN establishment is not significantly different from the waiting time to cancer. Here, CIN mutators become established by hitchhiking with SCNAs containing multiple beneficial mutation. As a result, an expanding mutator lineage may already carry enough beneficial mutations for the tumor to become cancerous as soon as it is established (as seen, for example, in Figure 1c) or can quickly acquire additional mutations in large clusters thereafter. On the other hand, in genomes with clustered deleterious loci, CIN mutators spread by hitchhiking with SCNAs generally containing only a single beneficial mutation. Consequently, even after CIN is established, multiple beneficial mutation must still sweep through the population before cancer evolves. Chromosomal instability mutators do, however, significantly accelerate cancer evolution by rapidly producing the necessary beneficial mutations (albeit still via SCNAs with only a single beneficial mutation each).

### 3.2 Oncogenic and tumor‐suppressive loci in the human genome are distributed with relatively high variance

Simulations show that increased variance in either beneficial or deleterious fitness effects of SCNAs, resulting from increased spatial clustering of fitness‐affecting mutations, can promote the evolution of CIN. To assess the variance in beneficial fitness effects of potential SCNAs in the human genome, we examined the spatial distribution of tumor suppressor and oncogene loci identified by the TUSON algorithm, developed by Davoli et al. (2013). Intriguingly, the work of Davoli et al. showed a significant relationship between the genomic distribution and potency of tumor suppressors and oncogenes (summarized as Chrom and Charm scores, see Methods) and patterns of chromosomal deletions and amplifications from sequenced tumors.

**Figure 4:**
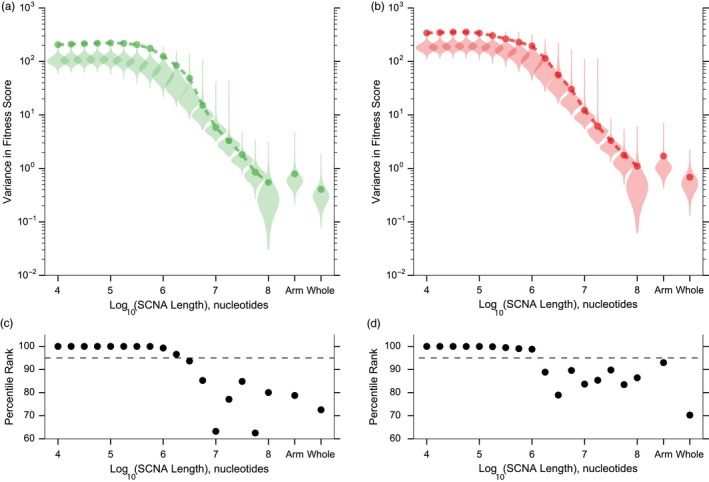
Oncogenes and tumors suppressors are distributed with relatively high variance in the human genome. Variance in the distributions of (a) oncogenic and (b) tumor‐suppressive effects of the human SCNAs (circles). Violin plots in a and b: Variances of 10,000 distributions generated by permutation (see Methods). Percentile ranks of observed variances in (c) oncogenic effect distributions and (d) tumor‐suppressive effect distributions among variances of permuted distributions (in panels a and b, respectively). Horizontal dashed line: 95th percentile rank

Using the methodology of their study, we evaluated variance in the distribution of oncogenic and tumor‐suppressive effects of potential human SCNAs of different size, including focal SCNAs shorter than a chromosome arm, as well as SCNAs the length of a chromosome arm and a whole chromosome (Methods). We observed that variance in the distributions of oncogenic (Figure 4a) and tumor‐suppressive (Figure 4b) effects was maximized for shorter SCNAs and decreased for longer SCNAs. We then evaluated the observed variance using a permutation approach (Methods). We found that the true variance in the distributions of both oncogenes and tumor suppressors was consistently higher than the mean variance of the permuted distributions across all of SCNA lengths examined and significantly higher (above the 95th percentile) for focal SCNAs shorter than ~10^6^ nucleotides. Thus, it appears that beneficial oncogenic and tumor‐suppressive effects are, in fact, more clustered in potential SCNAs than expected by chance, suggesting that the human genome could be organized in a way that could promote CIN hitchhiking.

### 3.3 Spatial organization of the genome affects the success of CIN‐inhibiting therapies

In simulations, we showed that high variance in the spatial distribution of beneficial loci across SCNAs can promote the evolution of CIN, which in turn can significantly accelerate carcinogenesis. Furthermore, our analysis of the distribution of known oncogenes and tumor suppressors suggested that mutations beneficial to neoplastic cells may be more clustered than random in the human genome. In light of these observations, we investigated whether the evolution of CIN could be inhibited by either increasing the mutation rate of CIN mutators or by exacerbating the effects of individual deleterious loci. Both therapeutic strategies have been previously shown to successfully reduce tumor size by exploiting its deleterious mutational load (McFarland et al., 2013). Correspondingly, we wanted to test whether these strategies could also inhibit CIN evolution by increasing the deleterious load associated with CIN mutators.

**Figure 5:**
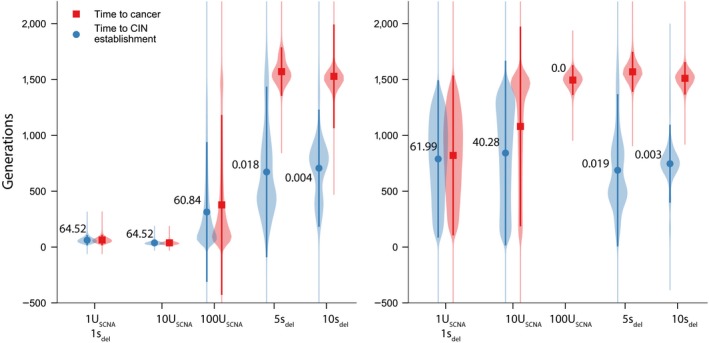
Exacerbating deleterious mutations is more effective at inhibiting CIN than increasing the rate of CIN. (a) In genomes with high clustering of beneficial mutations (beta‐binomially distributed, *µ* = 1). (b) In genomes with intermediate clustering of beneficial mutations (geometrically distributed, *µ* = 1). In both panels: deleterious mutations distributed with no clustering (Dirac delta, *µ* = 100). Circles are mean values calculated over 100,000 runs of simulation (error bars represent ±95% CI, all times are shown with violin plots). Model parameters as in Figures 2 and 3 except where noted. See Methods for distribution details. $P_{10\%}^{\mathrm{CIN}}/P_{10\%}^{\mathrm{neutral}}$values next to each data point

Using our model, we assessed the effect of increasing both CIN rate (*U*_SCNA_) and deleterious mutation effects (*s*_del_) in genomes characterized by high (beta‐binomially distributed, Figure 1c) and intermediate (geometrically distributed, Figure 1b) clustering of beneficial mutations. For the most clustered genomes (Figure 5a), increasing *U*_SCNA_ 10‐fold was completely ineffective at preventing either the establishment of CIN or rapid carcinogenesis. Increasing *U*_SCNA_ 100‐fold (to 1 SCNA per cell per generation) produced only a minor effect on the probability of establishment of CIN mutators, although both CIN establishment and carcinogenesis were somewhat delayed. Increasing *U*_SCNA_ was considerably more effective at inhibiting its establishment and minimizing its role in carcinogenesis in genomes characterized by intermediate clustering (Figure 5b). In this case, 100‐fold stronger CIN mutators were completely inhibited while the probability of establishment of 10‐fold stronger mutators was reduced by ~30%. On the other hand, increasing *s*_del_ only five‐fold resulted in CIN mutators becoming strongly disfavored by selection in both genomes, while the average waiting time to cancer increased to non‐CIN levels seen in Figure 3. Thus, while increasing CIN rate may successfully inhibit CIN evolution in some spatially clustered genomes, magnifying the effects of deleterious mutations appears to be a considerably more effective strategy. We speculate on the reasons for this difference below.

## 4 DISCUSSION

Here, we have developed a stochastic, individual‐based simulation model of clonal populations to examine the evolution of chromosomal instability (CIN) via indirect selection on associated beneficial variation. The propensity of genomic mutators to spread in clonal populations via indirect selection has been extensively studied in evolutionary theory (Gerrish, Colato, Perelson, & Sniegowski, 2007; Kimura, 1967; Taddei et al., 1997) and demonstrated in experimental microbial populations (Chao & Cox, 1983; Raynes & Sniegowski, 2014). Indirect selection on mutators and their potential role in carcinogenesis have also been investigated in computational and analytic models of cancer progression (Beckman & Loeb, 2006; Datta et al., 2013). However, whether indirect selection could favor CIN mutators during carcinogenesis has remained unclear. The reason is that CIN mutators generate SCNAs large enough to simultaneously affect multiple loci. As deleterious mutations generally outnumber beneficial ones, SCNAs with an overall beneficial effect may be too scarce to allow for CIN hitchhiking. Thus, we hypothesized that indirect selection could only favor CIN in genomes in which fitness‐affecting loci were distributed in a way that minimized the co‐occurrence of beneficial and deleterious loci in potential SCNAs.

In agreement with our hypothesis, simulations showed that the genomic distribution of fitness‐affecting loci can strongly influence indirect selection on CIN‐inducing mutators. CIN mutators failed to establish or affect carcinogenesis when both beneficial and deleterious loci were evenly distributed among potential SCNAs. However, spatial clustering of either the beneficial or the deleterious loci promoted rapid hitchhiking of CIN mutators and accelerated carcinogenesis. In genomes characterized by the higher clustering of the beneficial loci, CIN mutators succeeded by acquiring SCNAs containing multiple beneficial mutations, rather than acquiring such mutations individually (as previously seen in models of MSI mutators, (Datta et al., 2013)). On the other hand, in genomes characterized by the higher clustering of the deleterious loci, CIN mutators succeeded by acquiring SCNAs with few beneficial mutations but a reduced load of deleterious ones. Once established, CIN mutators in such genomes were able to accelerate carcinogenesis by rapidly producing additional beneficial mutations via further SCNAs.

Importantly, in our model, we assume that all single‐locus mutations have a constant effect on a cell's fitness. Therefore, the availability of beneficial SCNAs that could facilitate hitchhiking in simulation depended solely on the variance in the physical distribution of beneficial and deleterious loci. In a real tumor, however, different mutations will likely have different effects on a cell's fitness. As a result, the distribution of beneficial effects of real SCNAs will be determined by both the physical distribution of individual loci and the fitness distribution of their mutational effects. For example, the overall beneficial effect of an SCNA could be set by a cluster of smaller effect beneficial mutations or, instead, a single mutation of large effect. Hitchhiking of CIN mutators should then depend on the variance in the distribution of beneficial effects of potential SCNAs being sufficiently high to allow for SCNAs whose beneficial effects could compensate for their deleterious load.

Unfortunately, while many potential oncogenic and tumor‐suppressing loci have been discovered, little is known about their fitness effects as these have been difficult to measure empirically. Thus, as a first approximation of the variance in fitness of potential human SCNAs, we used the TUSON algorithm by Davoli et al. (2013), which ranks human loci based on the likelihood of their mutations acting as either oncogenes or tumor suppressors. Like Davoli and colleagues, we assigned each gene a fitness effect corresponding to its rank, resulting in a discrete uniform distribution of fitness effects. Given this simple scheme, both potential oncogenes and tumor suppressors appeared to be more spatially clustered than expected, with shorter SCNAs up to ~10^6^ nucleotides significantly so. Thus, the human genome may be organized in such a way that some of the available SCNAs have sufficiently large beneficial effects that overcome their deleterious load. If such SCNAs are available, our model suggests that alleles that induce CIN may be favored by indirect selection even in the absence of any direct benefit to a neoplastic cell's fitness. Furthermore, the relatively high clustering of selected loci across the shorter SCNAs suggests that shorter SCNAs are particularly likely to be positively selected during carcinogenesis and should thus be overrepresented in genomically unstable tumors. Intriguingly, a comprehensive survey of focal SCNA length across multiple cancer types by Beroukhim et al. (2010) showed an inverse relationship between SCNA length and frequency, with a median length of 1.8 × 10^6^ nucleotides (Beroukhim et al., 2010) Note that this observation is only consistent with the prediction that natural selection should favor shorter SCNAs and is not evidence for the role of indirect selection in CIN evolution.

It is surprising that the human genome may be organized in a way that promotes CIN evolution. After all, natural selection might have been expected to eliminate or at least reduce clustering of oncogenes and tumor suppressors to lower cancer susceptibility. However, it is important to note that such selection would have been only one of the determinants of genome organization. It is becoming well understood that eukaryotic genes are not randomly distributed across the genome. Related genes and gene families that have arisen through gene duplication may be expected to co‐localize (Demuth & Hahn, 2009). Genes with similar or coordinated expression are also frequently clustered (Hurst, Pál, & Lercher, 2004). Importantly, cancer genes identified by Davoli et al. (2013) and used in our analysis appear to be significantly enriched for a handful of functions expected to aid in carcinogenesis, such as cell‐cycle control and apoptosis. In light of this observation, it seems plausible that many cancer genes may be related due to their common origin by duplication or share combined regulation and are more clustered than expected as a result. For example, two tumor suppressors frequently inactivated in colorectal cancer, *SMAD2* and *SMAD4* (Fearon, 2011), belong to the same protein family and are located within several megabases of each other. Moreover, we would speculate that the evolution of reduced genomic clustering may be less likely than the evolution of other, perhaps more accessible, mechanisms to suppress cancer (such as additional tumor suppressor genes). As a result, genomic clustering of cancer genes may have persisted despite its potential role in CIN evolution and carcinogenesis.

Quantifying the true variance in fitness effects of potential human SCNAs requires precise fitness measurements of mutations beneficial to a neoplastic cell's fitness. Theoretical studies have produced estimates of mean effects of such mutations from ~0.4% to ~60% (Beerenwinkel et al., 2007; Bozic et al., 2010; McFarland et al., 2014). Vermeulen et al. (2013) were also able to empirically measure the selective effects of three mutations in *p53*,* APC,* and *Kras* in cells of a mouse intestine. Fitness contributions of mutations in most of the known oncogenic and tumor‐suppressive loci are yet to be estimated empirically. By considering the distribution of all known driver mutations discovered in different cancer types, we also have implicitly assumed such mutations would be equally beneficial in all cancers. However, the fitness contribution of a mutation in a particular locus should depend greatly on the selective environment and the potential interactions with other mutations present in the genome and will thus likely differ between cancer types. A more rigorous test of the susceptibility of the genome to CIN hitchhiking would correlate the distribution of beneficial mutations, given their actual fitness contributions in a particular cancer, with the rate of CIN in that cancer. The necessary data are, however, currently unavailable.

Understanding when and how CIN evolves by indirect selection and the potential role of genomic organization in CIN evolution can have important practical implications. While the necessity of CIN for cancer is debatable, our simulations show that once it evolves, CIN can rapidly produce beneficial variation and accelerate carcinogenesis. There is also some evidence that CIN may evolve early in cancer progression (Olaharski et al., 2006; Rajagopalan et al., 2004; Shih et al., 2001; Tonini, 2017). Thus, a potential therapy to prevent CIN evolution in the first place may be able to severely inhibit cancer development. Building on the earlier work of McFarland et al. (2013), we also used our model to examine whether increasing the rate of CIN or the cost of deleterious mutations could prevent evolution of CIN. Both therapeutic strategies aim to increase the deleterious mutational load and may theoretically be expected to inhibit mutator evolution. Intriguingly, we discovered that in genomes characterized by high variance in the distribution of beneficial mutations, increasing the deleterious effects of individual mutations was considerably more effective than increasing the rate of CIN. The disparity in the effectiveness of the two strategies is likely due to the mechanics of CIN hitchhiking in these genomes. Stronger CIN mutators can still produce the rare but very beneficial SCNAs available in these genomes, which allows them to rapidly spread despite the associated deleterious load. Correspondingly, in genomes characterized by lower variance, stronger CIN mutators become less successful. On the other hand, exacerbating the cost of deleterious mutations dramatically decreases the fitness effect of all available SCNAs, amplifying the deleterious load associated with any increase in the rate of CIN and effectively inhibiting CIN mutators.

In the study of McFarland et al. (2013), both increasing the overall mutation rate of a tumor and magnifying the effects of deleterious mutations successfully led to cancer regression. In their model, both strategies work by strengthening selection against deleterious mutations accumulated by neoplastic populations during carcinogenesis. However, magnifying the deleterious effects of these mutations proved to be a more effective therapy in simulation than increasing the mutation rate. Our results agree that exacerbating deleterious effects could also be a more effective strategy to prevent CIN evolution and slow carcinogenesis, assuming the potentially high clustering of carcinogenic beneficial mutations in the human genome. In the clinic, exacerbating deleterious mutations could be potentially achieved, as suggested by McFarland et al., by targeting cellular mechanisms that act to ameliorate their effects in newly made proteins; examples of such mechanisms include chaperones that may help proteins destabilized by deleterious mutations maintain activity (Karras et al., 2017; Rutherford & Lindquist, 1998) and proteosomes that degrade such proteins (Crawford, Walker, & Irvine, 2011).

In summary, extending earlier computational models of cancer progression, we have developed a new model incorporating both SCNA mutations and CIN‐inducing mutators to investigate the role of indirect selection in CIN evolution. Our model predicts that CIN mutators are strongly favored by indirect selection in genomes in which mutations that affect neoplastic fitness are clustered within potential SCNAs. Interestingly, preliminary examination of the distribution of human oncogenes and tumor suppressors suggests that human genomes may, indeed, be organized in such a way as to be susceptible to CIN hitchhiking via indirect selection, although more data on actual fitness contributions of these mutations are needed. Understanding whether CIN evolves by hitchhiking and the role that genome organization plays in CIN evolution may help in future therapeutic efforts aimed at selecting against CIN to inhibit carcinogenesis.

## CONFLICT OF INTEREST

None declared.

## DATA ARCHIVING

Julia code for the simulation and the simulated data sets are available at https://github.com/yraynes/Chromosomal-Instability.
