# Supplementary figures and images for: Genomic clustering of fitness‐affecting mutations favors the evolution of chromosomal instability
Source: Evol Appl. 2018 Oct 11;12(2):301–13. doi: 10.1111/eva.12717 (PMC6346662; doi:10.1111/eva.12717)

Generations

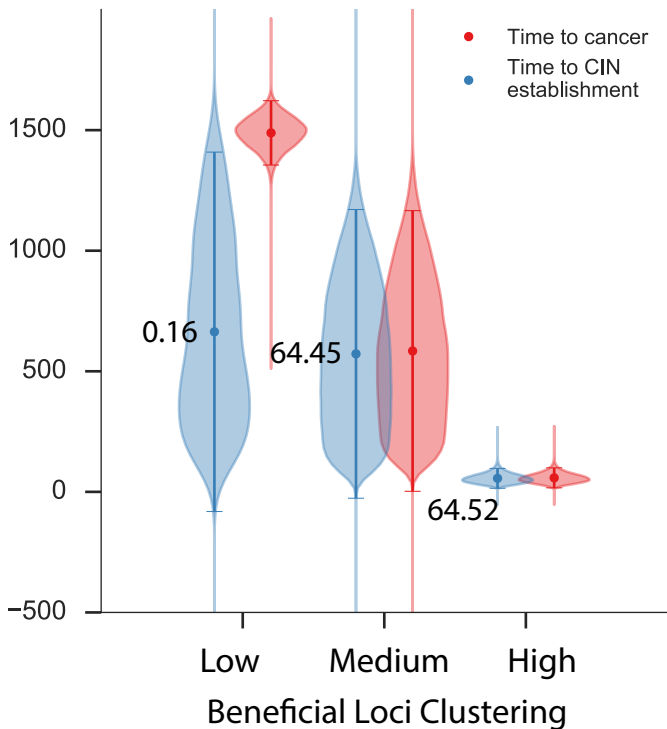

Supplement: Supplementary file 1 [file EVA-12-301-s001.pdf]

Generations

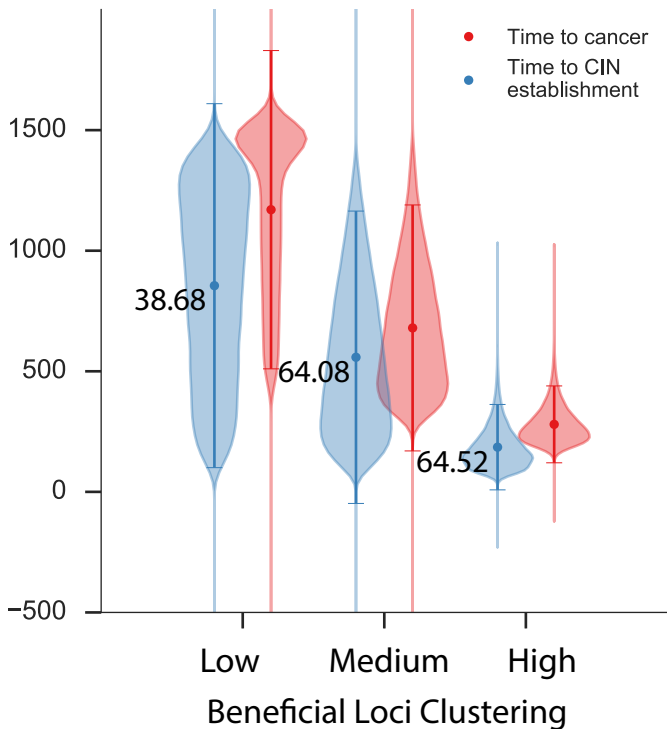

Supplement: Supplementary file 2 [file EVA-12-301-s002.pdf]
